# Editorial: Diagnosis and therapeutic interventions for oral diseases

**DOI:** 10.3389/froh.2025.1747983

**Published:** 2025-12-02

**Authors:** Subhabrata Maiti, Artak Heboyan

**Affiliations:** 1 Department of Prosthodontics, Saveetha Dental College and Hospitals, Saveetha Institute of Medical and Technical Sciences, Saveetha University, Chennai, India; 2 Department of Research Analytics, Saveetha Dental College and Hospitals, Saveetha Institute of Medical and Technical Sciences, Saveetha University, Chennai, India; 3 Department of Prosthodontics, Faculty of Stomatology, Yerevan State Medical University after Mkhitar Heratsi, Yerevan, Armenia

**Keywords:** precision oral healthcare, molecular diagnostics, transcriptomics, personalized therapies, immunomodulation

Advances in molecular diagnostics, computational modeling, and tailored therapies are driving a significant revolution in oral healthcare. Proactive, data-driven techniques that prioritize early detection, biological insight, and individualized care are replacing the long-standing model of treating disease only after clinical manifestation. This Research Topic, “*Diagnosis and Therapeutic Interventions for Oral Diseases*,” brings together five contributions that collectively illuminate this transition, illustrating how the integration of diagnostics and therapeutics is reshaping both scientific understanding and clinical management of oral conditions.

## Advances in diagnostic innovation

The improvement of diagnostic accuracy is a recurring theme throughout this Topic. Clinicians can now identify disease processes at earlier and more subtle stages, improving prognosis and enabling more targeted treatments.

In Czajka et al. the authors investigate the interplay between host health determinants and antifungal resistance patterns. Their findings highlight how local epidemiological and health factors shape infection profiles, emphasizing the clinical need for surveillance and personalized antifungal strategies.

At the molecular level, Chianca et al. demonstrates the utility of enhanced genomic amplification in increasing the sensitivity of bacterial detection. This approach refines microbiological diagnostics in endodontics, revealing subclinical bacterial persistence that could otherwise escape detection using standard PCR techniques. Such advancements align directly with the broader trend toward biologically informed, precision diagnostics in oral healthcare.

Complementing these developments, Natarajan and Umapathy highlights the transformative role of transcriptomic profiling in identifying key molecular drivers of malignancy. By delineating gene expression patterns associated with oral carcinogenesis, this work reinforces the importance of integrating omics data into both risk assessment and therapeutic design, establishing a foundation for future precision oncology applications within dentistry.

## Innovations in therapeutic and regenerative approaches

While diagnostic technology defines the first frontier of precision dentistry, equally significant progress is evident in therapeutic development. The case report Liu et al. presents a compelling example of individualized clinical management. Through coordinated interdisciplinary care, the authors demonstrate that even teeth once considered non-restorable may be salvaged when biologically informed decision-making and contemporary endodontic materials are employed. This case exemplifies how precision treatment planning, supported by advanced imaging and biomaterials, can redefine clinical expectations.

Therapeutic research also extends into the domain of host–pathogen modulation. In Alotaibi et al., investigators explore the immunomodulatory potential of combined pharmacologic therapy. Their study reveals synergistic effects on inflammatory responses, suggesting new avenues for managing oral and systemic inflammatory conditions. This work underscores the growing importance of translational research that bridges immunology and oral medicine, expanding the therapeutic toolkit beyond traditional antimicrobial strategies.

## Integration, translation, and future directions

Taken together, these findings depict an evolving landscape where therapeutic innovation and diagnostic precision are increasingly interconnected. Collectively, they highlight the emergence of a new paradigm in oral healthcare that emphasizes biological individuality, early detection, and prevention. This Topic illustrates the scope and complexity of contemporary scientific research in oral medicine—from the genetic characterization of infectious and neoplastic diseases to the regeneration of damaged tissues and the optimization of immune responses.

The focus on translational relevance is equally important. Each study demonstrates how laboratory discoveries can evolve into practical tools and procedures for clinicians, bridging the gap between bench research and clinical practice. The research in this collection exemplifies the real-world application of innovation to patient care, whether through enhancing diagnostic precision, improving antimicrobial stewardship, or reframing clinical decision-making.

## Conclusion

The contributions to “*Diagnosis and Therapeutic Interventions for Oral Diseases”* collectively affirm that the future of oral healthcare lies in integration—across technologies, disciplines, and biological systems. These studies demonstrate how tailored therapies and precision diagnostics can work synergistically to enhance patient outcomes from molecular, cellular, and clinical perspectives. As dentistry moves toward a more predictive and personalized model, ongoing collaboration among researchers, clinicians, and educators will be essential to ensure that innovation translates into accessible, patient-centered care.

The next generation of precision oral healthcare will be defined by the convergence of diagnostics and therapeutics, and this Research Topic serves as both a snapshot of current advancements and a foundation for future exploration.

